# Supplementary figures and images for: Association between estimated plasma volume status and acute kidney injury in patients who underwent coronary revascularization: A retrospective cohort study from the MIMIC-IV database
Source: PLoS One. 2024 Jun 12;19(6):e0300656. doi: 10.1371/journal.pone.0300656 (PMC11168641; doi:10.1371/journal.pone.0300656)

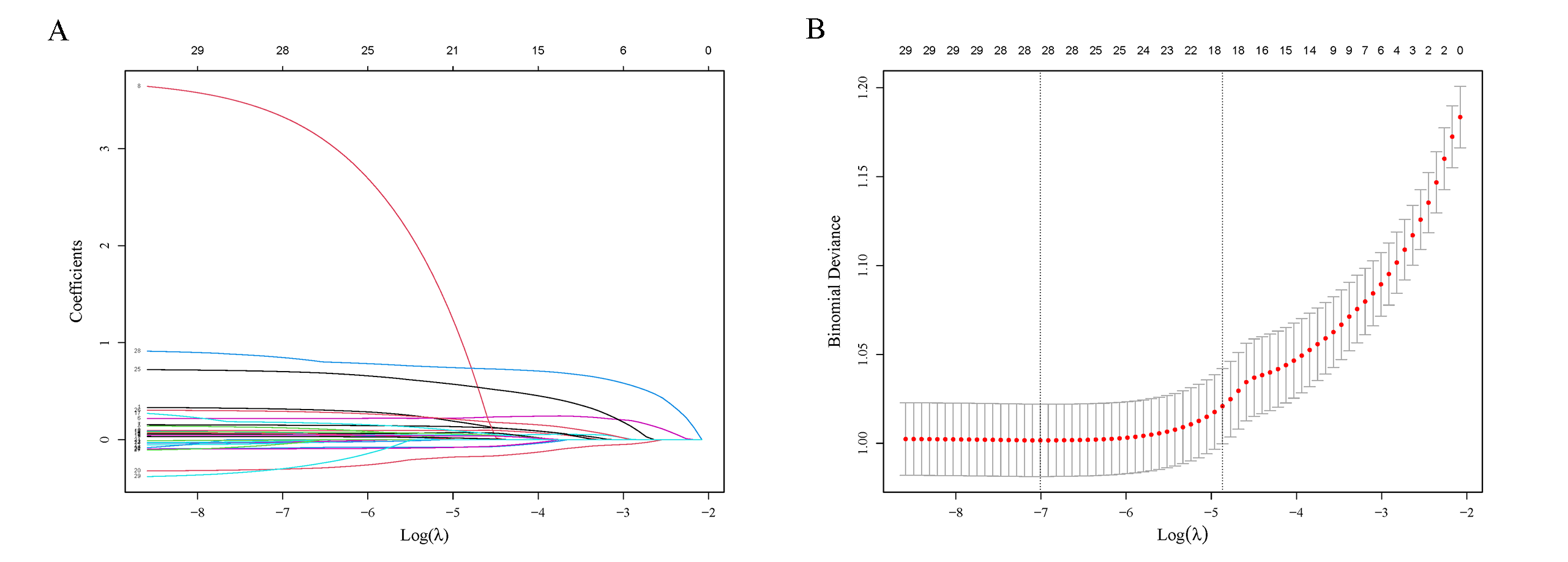

Supplement: S1 Fig — A: Path plot of Lasso coefficient; B: Lasso cross-validation plot. (TIF) [file pone.0300656.s001.tif]
