# Supplementary material for: Association between estimated plasma volume status and acute kidney injury in patients who underwent coronary revascularization: A retrospective cohort study from the MIMIC-IV database
Source: PLoS One. 2024 Jun 12;19(6):e0300656. doi: 10.1371/journal.pone.0300656 (PMC11168641; doi:10.1371/journal.pone.0300656)
Supplement: S1 Table — (DOCX) [file pone.0300656.s003.docx]

Table S1 Sensitivity analyses before and after imputation of missing data

| Variables | After (N=4062) | Before (N=4062) | Statistics | *P* |
| --- | --- | --- | --- | --- |
| GCS, score, Mean ± SD | 13.44 ± 3.46 | 13.44 ± 3.46 | t = 0.005 | 0.996 |
| Sodium, mEq/L, Mean ± SD | 135.62 ± 3.06 | 135.62 ± 3.06 | t = -0.010 | 0.992 |
| Potassium, mEq/L, Mean ± SD | 4.61 ± 0.76 | 4.62 ± 0.76 | t = -0.008 | 0.994 |
| 24 h urine output, ml, M(Q₁, Q₃) | 1845.00 (1359.00-2475.00) | 1845.00 (1358.00-2475.00) | W = 7842139.5 | 0.989 |
| Heart rate, bpm, Mean ± SD | 80.62 ± 11.30 | 80.63 ± 11.30 | t = -0.012 | 0.990 |
| SBP, mmHg, Mean ± SD | 114.17 ± 18.39 | 114.17 ± 18.40 | t = -0.001 | 0.999 |
| DBP, mmHg, Mean ± SD | 60.43 ± 12.42 | 60.43 ± 12.42 | t = 0.018 | 0.985 |
| Glucose, mg/dL, M(Q₁, Q₃) | 135.00 (114.00-164.00) | 135.00 (114.00-164.00) | W = 7842927 | 0.983 |
| Calcium, mmol/L, Mean ± SD | 2.38 ± 2.74 | 2.38 ± 2.74 | t = 0.019 | 0.985 |
| Anion gap, mEq/L, Mean ± SD | 11.70 ± 3.08 | 11.70 ± 3.08 | t = 0.022 | 0.982 |
| PTT, sec, M(Q₁, Q₃) | 30.40 (27.30-35.30) | 30.40 (27.30-35.30) | W = 7657084 | 0.972 |
| INR, M(Q₁, Q₃) | 1.40 (1.20-1.50) | 1.40 (1.20-1.50) | W = 7648911 | 0.970 |
| PT, sec, M(Q₁, Q₃) | 15.10 (13.80-16.70) | 15.10 (13.80-16.70) | W = 7641843 | 0.930 |
| PH, units, Mean ± SD | 7.40 ± 0.07 | 7.40 ± 0.07 | t = 0.213 | 0.832 |
| Temperature, ℃, Mean ± SD | 36.32 ± 0.62 | 36.32 ± 0.62 | t = -0.099 | 0.921 |
| Lactate, mmol/L, M(Q₁, Q₃) | 2.10 (1.60-2.70) | 2.10 (1.60-2.70) | W = 6895495.5 | 0.785 |
| PaCO_2_, mmHg, Mean ± SD | 40.99 ± 6.34 | 40.99 ± 6.40 | t = 0.000 | 1.000 |
| PaO_2_, mmHg, Mean ± SD | 306.06 ± 105.43 | 305.78 ± 104.73 | t = 0.116 | 0.908 |
| Respiratory rate, insp/min, M(Q₁, Q₃) | 15.00 (14.00-17.00) | 15.00 (14.00-17.00) | W = 6783804.5 | 0.901 |

GCS: glasgow coma scale; SBP: systolic blood pressure; DBP: diastolic blood pressure; PTT: partial thromboplastin time; INR: international normalized ratio; PT: prothrombin time; PH: pondus hydrogenii; t: t-test; W: Wilcoxon rank sum test;
