# Supplementary material for: Association between estimated plasma volume status and acute kidney injury in patients who underwent coronary revascularization: A retrospective cohort study from the MIMIC-IV database
Source: PLoS One. 2024 Jun 12;19(6):e0300656. doi: 10.1371/journal.pone.0300656 (PMC11168641; doi:10.1371/journal.pone.0300656)
Supplement: S2 Table — (DOCX) [file pone.0300656.s004.docx]

Table S2 The collinearity tests between covariates

| Variables | VIF |
| --- | --- |
| Age | 1.2261 |
| Race | 1.0113 |
| Blood disease | 1.0306 |
| SAPSII | 1.1589 |
| CCI | 1.1885 |
| RDW-CV | 1.0929 |
| eGFR | 1.1670 |
| INR | 1.0400 |
| Glucose | 1.0591 |
| Calcium | 1.3465 |
| pH | 1.2429 |
| PaCO_2_ | 1.2247 |
| Mechanical ventilation, | 1.0927 |
| Vasopressors | 1.1555 |
| Diuretic | 1.0427 |
| Weight | 1.1595 |

SAPSII: simplified acute physiology score II; CCI: charlson comorbidity index; RDW-CV: red blood cell distribution width; eGFR: estimated glomerular filtration rate; INR: international normalized ratio; pH: pondus hydrogenii.
