# Supplementary material for: Association between estimated plasma volume status and acute kidney injury in patients who underwent coronary revascularization: A retrospective cohort study from the MIMIC-IV database
Source: PLoS One. 2024 Jun 12;19(6):e0300656. doi: 10.1371/journal.pone.0300656 (PMC11168641; doi:10.1371/journal.pone.0300656)
Supplement: S3 Table — (DOCX) [file pone.0300656.s005.docx]

Table S3 Comorbidities of CCI in AKI and non-AKI

| Variables | Total (N=3961) | Non-AKI (N=1098) | AKI (N=2863) | Statistics | *P* |
| --- | --- | --- | --- | --- | --- |
| Myocardial infarct, n (%) |  |  |  | χ² = 8.307 | 0.004 |
| No | 2114 (53.37) | 545 (49.64) | 1569 (54.8) |  |  |
| Yes | 1847 (46.63) | 553 (50.36) | 1294 (45.2) |  |  |
| Congestive heart failure, n (%) |  |  |  | χ² = 32.887 | <0.001 |
| No | 2912 (73.52) | 879 (80.05) | 2033 (71.01) |  |  |
| Yes | 1049 (26.48) | 219 (19.95) | 830 (28.99) |  |  |
| peripheral_vascular_disease, n (%) |  |  |  | χ² = 10.703 | 0.001 |
| No | 3455 (87.23) | 989 (90.07) | 2466 (86.13) |  |  |
| Yes | 506 (12.77) | 109 (9.93) | 397 (13.87) |  |  |
| Cerebrovascular disease, n (%) |  |  |  | χ² = 10.772 | 0.001 |
| No | 3555 (89.75) | 1014 (92.35) | 2541 (88.75) |  |  |
| Yes | 406 (10.25) | 84 (7.65) | 322 (11.25) |  |  |
| Dementia, n (%) |  |  |  | χ² = 0.097 | 0.756 |
| No | 3935 (99.34) | 1092 (99.45) | 2843 (99.3) |  |  |
| Yes | 26 (0.66) | 6 (0.55) | 20 (0.7) |  |  |
| Chronic pulmonary disease, n (%) |  |  |  | χ² = 2.655 | 0.103 |
| No | 3179 (80.26) | 900 (81.97) | 2279 (79.6) |  |  |
| Yes | 782 (19.74) | 198 (18.03) | 584 (20.4) |  |  |
| Rheumatic disease, n (%) |  |  |  | χ² = 2.567 | 0.109 |
| No | 3833 (96.77) | 1071 (97.54) | 2762 (96.47) |  |  |
| Yes | 128 (3.23) | 27 (2.46) | 101 (3.53) |  |  |
| Peptic ulcer disease, n (%) |  |  |  | χ² = 0.324 | 0.569 |
| No | 3925 (99.09) | 1086 (98.91) | 2839 (99.16) |  |  |
| Yes | 36 (0.91) | 12 (1.09) | 24 (0.84) |  |  |
| Liver disease, n (%) |  |  |  | χ² = 0.047 | 0.828 |
| No | 3818 (96.39) | 1060 (96.54) | 2758 (96.33) |  |  |
| Yes | 143 (3.61) | 38 (3.46) | 105 (3.67) |  |  |
| Diabetes, n (%) |  |  |  | χ² = 16.334 | <0.001 |
| No | 2380 (60.09) | 716 (65.21) | 1664 (58.12) |  |  |
| Yes | 1581 (39.91) | 382 (34.79) | 1199 (41.88) |  |  |
| Malignant tumor, n (%) |  |  |  | χ² = 1.963 | 0.161 |
| No | 3837 (96.87) | 1071 (97.54) | 2766 (96.61) |  |  |
| Yes | 124 (3.13) | 27 (2.46) | 97 (3.39) |  |  |
| Paraplegia, n (%) |  |  |  | χ² = 0.970 | 0.325 |
| No | 3937 (99.39) | 1094 (99.64) | 2843 (99.3) |  |  |
| Yes | 24 (0.61) | 4 (0.36) | 20 (0.7) |  |  |
| No |  |  |  | χ² = 11.788 | 0.001 |
| Yes | 3334 (84.17) | 960 (87.43) | 2374 (82.92) |  |  |
| No | 627 (15.83) | 138 (12.57) | 489 (17.08) |  |  |
| Aids, n (%) |  |  |  | - | 0.672 |
| No | 3955 (99.85) | 1096 (99.82) | 2859 (99.86) |  |  |
| Yes | 6 (0.15) | 2 (0.18) | 4 (0.14) |  |  |

AKI: acute kidney injury; CCI: Charlson comorbidity index; χ²: Chi-square test; -: Fisher exact.
