# Supplementary material for: Association between estimated plasma volume status and acute kidney injury in patients who underwent coronary revascularization: A retrospective cohort study from the MIMIC-IV database
Source: PLoS One. 2024 Jun 12;19(6):e0300656. doi: 10.1371/journal.pone.0300656 (PMC11168641; doi:10.1371/journal.pone.0300656)
Supplement: S4 Table — (DOCX) [file pone.0300656.s006.docx]

Table S4 The ePVS level in different subgroups

| Subgroups | ePVS | Statistics | *P* |
| --- | --- | --- | --- |
| Age ≥65 |  | t' = -13.098 | <0.001 |
| No | 6.59 ± 2.10 |  |  |
| Yes | 7.56 ± 2.47 |  |  |
| Surgery |  | t' = -20.968 | <0.001 |
| PCI | 5.57 ± 1.95 |  |  |
| CABG | 7.47 ± 2.34 |  |  |
| Anticoagulation agents |  | t' = 19.605 | <0.001 |
| No | 7.45 ± 2.34 |  |  |
| Yes | 5.56 ± 2.00 |  |  |
| SOFA |  | t' = -11.205 | <0.001 |
| <5 | 6.76 ± 2.17 |  |  |
| ≥5 | 7.59 ± 2.50 |  |  |

PCI: percutaneous coronary intervention; CABG: coronary angioplasty bypass grafting; SOFA: sequential organ failure assessment; ePVS: estimated plasma volume status; t': satterthwaite t-test.
