# Supplementary material for: Association between estimated plasma volume status and acute kidney injury in patients who underwent coronary revascularization: A retrospective cohort study from the MIMIC-IV database
Source: PLoS One. 2024 Jun 12;19(6):e0300656. doi: 10.1371/journal.pone.0300656 (PMC11168641; doi:10.1371/journal.pone.0300656)
Supplement: Attachment — Submitted filename: Response to Reviewers.docx [file pone.0300656.s007.docx]

**Dear Seunghwa Lee**

Thanks a lot for your letter and comments on our manuscript. We have revised the manuscript and given point-by-point responses according to the editor and reviewer comments, which have been highlighted in red. We hope that the revised manuscript is now acceptable for publication in your journal.
Thanks again for what you have done for us and looking forward to your reply soon.

Best regards,

Xinping Yang

Reviewers' comments:

Reviewer's Responses to Questions

**Comments to the Author:**

Please use the space provided to explain your answers to the questions above. You may also include additional comments for the author, including concerns about dual publication, research ethics, or publication ethics. (Please upload your review as an attachment if it exceeds 20,000 characters)
1. Is the manuscript technically sound, and do the data support the conclusions?

The manuscript must describe a technically sound piece of scientific research with data that supports the conclusions. Experiments must have been conducted rigorously, with appropriate controls, replication, and sample sizes. The conclusions must be drawn appropriately based on the data presented.

Reviewer #1: Yes

Reviewer #2: Yes

Reviewer #3: Yes

Reply: Thanks a lot for your comments.

2. Has the statistical analysis been performed appropriately and rigorously？

Reviewer #1: Yes

Reviewer #2: Yes

Reviewer #3: N/A

Reply: Thanks a lot for your comments.

3. Have the authors made all data underlying the findings in their manuscript fully available?

The PLOS Data policy requires authors to make all data underlying the findings described in their manuscript fully available without restriction, with rare exception (please refer to the Data Availability Statement in the manuscript PDF file). The data should be provided as part of the manuscript or its supporting information, or deposited to a public repository. For example, in addition to summary statistics, the data points behind means, medians and variance measures should be available. If there are restrictions on publicly sharing data—e.g. participant privacy or use of data from a third party—those must be specified.

Reviewer #1: Yes

Reviewer #2: Yes

Reviewer #3: No

Reply: Thanks a lot for your comments. We have presented all our results in the revised manuscript or in the supplement tables.

4. Is the manuscript presented in an intelligible fashion and written in standard English?

PLOS ONE does not copyedit accepted manuscripts, so the language in submitted articles must be clear, correct, and unambiguous. Any typographical or grammatical errors should be corrected at revision, so please note any specific errors here.

Reviewer #1: Yes

Reviewer #2: No

Reviewer #3: No

Reply: Thanks a lot for your comments. We have carried out a proofreading of the full manuscript.

Reviewer #1: Thank you for the opportunity to review this manuscript. This was a retrospective cohort study using MIMIC-4 database. The authors demonstrated the association between estimated plasma volume status (ePVS) and the risk of AKI in patients undergoing coronary revascularization, which provides a promising parameter to evaluate the risk stratification management of ICU patients who underwent coronary revascularization. The study was generally well designed; However, there are a few additional points that need to be checked.

Major concern
1.As potential confounders, patients with anemia or polycythemia in the eligibility criteria are excluded or recognized.

Reply: Thanks a lot for your careful review of our manuscript. Data about anemia or polycythemia of study population in the MIMIC-IV were extracted. Finally, we discovered that fewer patients had polycythemia, so we included polycythemia as one of the exclusion population criteria and patients with polycythemia were excluded from this study. Given the large number of patients with anemia, anemia was considered as a confounding variable and used in subsequent covariate screening.

The sample size of the study was changed due to the changes of the inclusion and exclusion criteria. Here we present the final study population screening flowchart and related results.
2.Multi-factor regression must be comprehensively addressed. Does the equation indeed only consider the ePVS in its final form?

Reply: Thanks a lot for your comments. Plasma volume state (PVS) and ePVS were considered in our revised study. The PVS was calculated based on the patient’s body weight and hematocrit. Then, a percentage between the ideal plasma volume (iPV) and PV can be derived, and the difference in this percentage was referred to as the PVS. In the revised manuscript, we also used PVS as an exposure factor in our study to explore its association with the risk of AKI among patients after coronary revascularization. The results showed that there was no relationship between PVS and the risk of AKI among patients after coronary revascularization. Compared to PVS, ePVS can more accurately predict the risk of AKI among patients after coronary revascularization.
3.When conducting the regression analysis, it is important to consider collinearity, as there are multiple variables involved in the analysis.

Reply: Thanks a lot for your comments. We evaluated the collinearity between individual covariates, and we performed collinearity tests for included covariates. The variance inflation factor (VIF) values of each variable were less than 10, indicating that there was no high collinearity between these variables. Supplement table 2 presents the results for collinearity.

- 4.The t-test and chi-square test value are not commonly used in statistical presentation. Some measures in the table are presented as medians, while others are presented as means?

Reply: Thanks a lot for your comments. After carefully reviewing our manuscript, we took your advice and removed the statistic from the Table 1 of characteristics of the study population. Continuous variables with normal distribution were described as mean ± standard deviation (SD) and categorical variables were represented as number and percentage.

- 5.The basis for sub-group grouping can be represented using a forest map.

Reply: Thanks a lot for your comments. The sub-group grouping was depicted in the form of forest map in Figure 3.

- 6.The discussion section should be revised to enhance its clinical relevance.

Reply: Thanks a lot for your comments. We supplemented the clinical significance of ePVS in predicting the risk of AKI among patients after coronary revascularization. Previously, plasma volume was usually measured by tracer dilution techniques, and in the context of old age, high comorbidity burden, and economic problems, this invasive outpatient monitoring may not be widely available to all coronary artery diseases patients. The ePVS based on changes in serial hemoglobin/hematocrit measurements could represent a low-cost and easy to use alternative, which may be a potential prognostic indicator for the pre-bed management of patients who received coronary revascularization. Monitoring the ePVS level in patients who underwent coronary revascularization can help clinicians early identify patients with high risk of AKI and provide certain reference for risk stratification management and early intervention treatment of these patients.

Reviewer #2: Dear Author, thank you for the interesting article.

My clarifications are as follows:
1. Do clarify if the formula used to estimate the ePVS is the only formula available. Are there formulas that correct for gender differences and/or normalise for weight and height. If so, why was the formula in the study chosen as 1-Hct/Hb?

Reply: Thanks a lot for your comments. The PVS and ePVS were considered in our study. The PVS was calculated based on the patient’s body weight and hematocrit. The association between PBS and the risk of AKI in patients who underwent the coronary revascularization was evaluated, and the results showed no statistical association between PVS and the risk of AKI in patients who underwent the coronary revascularization. The ePVS was calculated by Duarte et al. to estimate the PV derived from Strauss’s formula using plasma volume measurements derived “instantaneous” as a single time point [1]. However, clinically, PVS is usually measured by tracer dilution techniques, but these methods are expensive, time-consuming and complicated. Therefore, ePVS based on hemoglobin and hematocrit calculations have been proposed and confirmed by Duarte et al. [1] as an alternative to radioisotope tracer detection. Although there was no statistical association between PVS and the risk of AKI in patients who underwent the coronary revascularization in our study population, future large-scale studies are needed to confirm the role of PVS as a clinically important indicator of poor prognosis in coronary artery disease.

1.	Duarte, K., et al., *Prognostic Value of Estimated Plasma Volume in Heart Failure.* JACC Heart Fail, 2015. **3**(11): p. 886-93.

2. I have concerns that hematocrit and hemoglobin can be acutely affected by many issues that may not be directly linked to volume status. Is there data on proportion of patients who have hematological conditions, erythropoietin stimulating agents that can affect your ePVS calculation?

Reply: Thanks a lot for your comments. Data of the patients with blood diseases based on ICD-9 codes with 280-289 and ICD-10 codes with D50-D89 were extracted from the MIMIC-IV database and presented in Table 1. Among 3,961 patients, 2,083 (52.59%) had blood diseases. Due to the limitation of the MIMIC-IV database, there was no contain data on the treatment of erythropoietin stimulating agents. Given that the iron uses may affect the results of ePVS calculation, we replace the erythropoietin stimulating agents with iron use, also presented in Table 1.

3. The incidence of AKI post coronary revascularization can also be related to other factors such as overall cardiac function. A patient with poorer cardiac function such as low EF maybe at higher risk of AKI (part of cardio-renal syndrome) and correspondingly more likely to be overloaded and so have higher risk of AKI but I noted that there is no data on the EF in the data set. Also the amount of contrast given during coronary revascularization is not known. Are these data available to be included?

Reply: Thanks a lot for your comments. Unfortunately, there were no data on EF in MIMIC-IV database, and only one patient had the amount of contrast. In view of the potential impact of these two factors on the risk of AKI after coronary revascularization, we consider them as the limitation of our study. In the future, larger cohort studies incorporating EF and the amount of contrast are needed to explore the association between ePVS and the risk of AKI in patients who underwent the coronary revascularization.

4. Is there any interaction between the ePVS and other dependent covariates that is strongly associated with AKI such as age and high SOFA scores?

Reply: Thank you for your comments. The interaction between ePVS and age and high SOFA scores were evaluated. As depicted in Table S4, the results showed that there was no interaction between ePVS and age and SOFA.

5. Minor grammatical error - page 5 line 70 , "to the best" and not beast.
Reply: Thank you for your careful review of our manuscript. We apologize for this error and have corrected it in the manuscript.

Reviewer #3: 1. Please explain when the patient received fluid resuscitation (plasma volume status were estimated) and when AKI was diagnosed?

Reply: Thank you for your careful review of our manuscript. Plasma volume status was estimated by measurement of hematocrit and hemoglobin within 24 hours after ICU admission. AKI was diagnosed during the patients admitted in the ICU. The median follow-up time was 0.86 (0.60, 1.25) days.
2. The author(s) should discuss why the high ePVS was associated with a risk of AKI in patients aged <65 years, but not in patients aged ≥65?

Reply: Thank you for your comments. The inclusion and exclusion criteria were revised according to the recommendations of the aforementioned reviewers. We reexamined the odds of ePVS and AKI among patients who underwent coronary revascularization based on age, surgery, anticoagulation agents and SOFA score. Finally, the results showed that regardless of whether the patients aged ≥65 years were all associated with a higher odds of AKI in patients who underwent coronary revascularization. Our study suggested that ePVS may have the ability to predict the odds of AKI in patients who underwent the coronary revascularization.
3. Is there any differences in ePVS between patients aged <65 and ≥ 65 years old?

Reply: Thank you for your comments. We compared the ePVS between patients aged <65 years old and ≥65 years old and found that the ePVS level was different between the two age groups (*P*<0.001). The results were presented in Table S4.
4. What were the comorbidities in patients with and without AKI respectively?

Reply: Thanks a lot for your comments. We explored the comorbidities in patients with and without AKI and presented the results in supplement table 3.
5. The English needs to be improved.

Reply: Thanks a lot for your comments. We have carried out a proofreading of the full manuscript.

6. PLOS authors have the option to publish the peer review history of their article ([what does this mean?](https://journals.plos.org/plosone/s/editorial-and-peer-review-process#loc-peer-review-history)). If published, this will include your full peer review and any attached files.


If you choose “no”, your identity will remain anonymous but your review may still be made public.


**Do you want your identity to be public for this peer review?** For information about this choice, including consent withdrawal, please see our [Privacy Policy](https://www.plos.org/privacy-policy).

Reviewer #1: **Yes:** Ming Wu

Reviewer #2: No

Reviewer #3: **Yes:** Feng SHEN
